# The Moral Impact of the COVID-19 Pandemic on Nurses’ Burnout, Work Satisfaction and Adaptive Work Performance: The Role of Autobiographical Memories of Potentially Morally Injurious Events and Basic Psychological Needs

**DOI:** 10.3390/ijerph19137645

**Published:** 2022-06-22

**Authors:** Mihaela Alexandra Gherman, Laura Arhiri, Andrei Corneliu Holman, Camelia Soponaru

**Affiliations:** Faculty of Psychology and Education Sciences, Alexandru Ioan Cuza University, Str. Toma Cozma 3, 700554 Iasi, Romania; laura.arhiri@uaic.ro (L.A.); andrei.holman@uaic.ro (A.C.H.); camelia.soponaru@uaic.ro (C.S.)

**Keywords:** potentially morally injurious event (PMIE), work satisfaction, autonomy, work motivation, COVID-19 pandemic, adaptive performance, nurses, burnout, episodic memories, self-determination theory

## Abstract

The COVID-19 pandemic resulted in unprecedented exposure to Potentially Morally Injurious Events (PMIEs) for nurses, in which they were both moral transgressors and moral victims, with deleterious consequences on their psycho-social health and functioning. Our experimental design compared memories of PMIEs with memories of severe moral transgressions (SMTs), in which participants were only moral transgressors. Drawing from Self-Determination Theory and research on moral auto-biographical episodic memories, we assessed a conceptual model describing the impact of recalling a single PMIE or SMT event on nurses’ burnout, work satisfaction and adaptive performance. Our convenience sample comprised 614 Romanian nurses, and data was analyzed with path analysis, general linear modelling, and *t*-tests. Findings showed that memories of PMIEs, compared to SMTs, were more autonomy thwarting, being associated with more controlled work motivation, less moral learning, higher burnout, less work satisfaction, and adaptive performance. Burnout, moral learning, and work satisfaction were significant mediators of the relationships between PMIE and SMT recall and, respectively, adaptive performance. Our results highlight the urgency for organizational practices of moral repair for nurses after the pandemic, along with interventions meant to increase their autonomy and self-determined work motivation.

## 1. Introduction

### 1.1. Potentially Morally Injurious Events in Healthcare during the COVID-19 Pandemic

During the COVID-19 pandemic, resource scarcity led to work-related ethical challenges which affected healthcare providers’ work performance, psychological health, and functionality [1,2,3]. The necessity of making very difficult, often life and death decisions about resource prioritizing resulted in frequent exposure to PMIEs [2,3,4,5]. PMIEs are severe moral transgressions for which the individual feels responsible, either because they perpetrated them or because they stood by passively and failed to act according to their moral principles [2]. PMIEs are associated with experiencing victimization and sometimes trauma, due to the perceived inability to act differently under circumstantial constraints [1,2,3,4,5]. Moral injury is the result of exposure to PMIEs, although not all exposure to PMIEs results in moral injury, it also has other detrimental consequences on psycho-social health and functioning [2].

### 1.2. Autobiographical Episodic Memories of Moral Transgressions

Autobiographical episodic memories of moral transgressions represent vivid, emotional, and detailed encodings of moral violations inflicted, witnessed, or suffered by the individual. Recent research suggests that there are differences in how vividly and readily we recall these memories according to our perceived roles of moral perpetrators or moral victims [6]. Also, the more severe the moral transgression, the better we recall it, especially when we perpetrated it [7]. This seems to occur even though recalling severe, self-perpetrated moral violations are accompanied by more intense negative affect and psychological distress than recalling more minor ones [7,8]. To the best of our knowledge, there are no studies that examine the effects of recalling memories of moral transgressions during which people perceived themselves as both victims and perpetrators [2], such as PMIEs.

### 1.3. Autonomy Thwarting in Work-Related, Autobiographical Episodic Memories

According to Self-Determination Theory (SDT) [9,10], workers’ motivation, thriving, and performance depend on the satisfaction/thwarting of three basic psychological needs: autonomy, the need to feel volitionally authentic in your behavior; competence, the need to feel efficient and effectual; relatedness, the need to feel connected to work colleagues [10]. Episodic memories of specific, non-repeated work-related events during which either of these three needs were thwarted can have a unique and significant impact on motivation, work satisfaction, and burnout, for up to two years [11,12]. The more thwarted the need(s), the more likely it is that the episodic memory becomes a self-defining memory [11]. Self-defining memories represent enduring concerns or unresolved conflicts, central and important to personal identity, which guide our behavior and shape our representations, all the while influencing occupational well-being and performance [11,13].

### 1.4. Work Motivation

According to the basic psychological need satisfaction experienced at work, motivation can be self-determined or controlled [9,10]. When workers’ needs are met, their motivation is self-determined: they perform their activities out of enjoyment, feeling that their job fits their values and identity, and that they exercise freedom in choosing their actions [9]. When their needs are thwarted, motivation becomes controlled, and workers perform duties out of guilt, external pressure, or for rewards/to avoid punishment [9,11].

### 1.5. Moral Learning following Moral Memory Recall

By and large, people consider themselves moral, and they need to feel and define themselves as such [8]. This moral identity is threatened by the frequent recall of their most severe moral violations [7]. However, recent studies found that remembering our more severe moral transgressions is followed by the simulation of morally upward counterfactuals—alternative moral courses of action (i.e., moral learning) [7]. The functional explanation of this phenomenon is that it represents how we learn from our past mistakes, especially since morally upward counterfactuals are followed by increased intentions to behave morally better in similar future situations [7]. Autonomy is a self-regulatory mechanism for moral learning: when autonomy is low, the number and self-regulatory quality of counterfactuals are reduced, leading to helplessness rather than learning and improvement [14].

### 1.6. Burnout

During the COVID-19 pandemic, nurses experienced more burnout than other health workers [15]. The burnout syndrome describes a loss of interest in work, a sense of hopelessness, depersonalization, and exhaustion [16]. In nurses, burnout jeopardized patient recovery more than any other factors during the COVID-19 pandemic [17]. It also contributed greatly to decreased productivity, mental, and physical health [18].

### 1.7. Work Satisfaction

Work satisfaction describes the degree to which people like their jobs and find them personally satisfying [19]. The pandemic decreased nurses’ work satisfaction [20], which led to high turnover intentions and poor quality of care, leading researchers to urge relevant stakeholders to take immediate action so that nurses feel supported organizationally in this regard [19].

### 1.8. Adaptive Performance

During the COVID-19 pandemic, nurses’ adaptive performance was an essential employee resource in managing the day-to-day crisis situations [21]. Adaptive performance was defined as “task-performance-directed behaviors individuals enact in response to or anticipation of changes relevant to job-related tasks” [22] (pp. 54–55). It measures the extent to which workers learn and apply new practices and strategies when confronted with (un)anticipated changes that may affect work outcomes.

### 1.9. Current Study

The COVID-19 pandemic inflicted an invisible epidemic of moral injury among healthcare providers, affecting nurses the most [1,2]. Moral injury was associated with secondary traumatic stress, generalized anxiety, depression, suicidal ideation, post-traumatic stress disorder, burnout, stress, and turnover intentions [23]. First, we must decrease the negative impact of potentially morally injurious events (PMIEs) on nurses’ mental health and performance [3,4].

**Figure 1:**
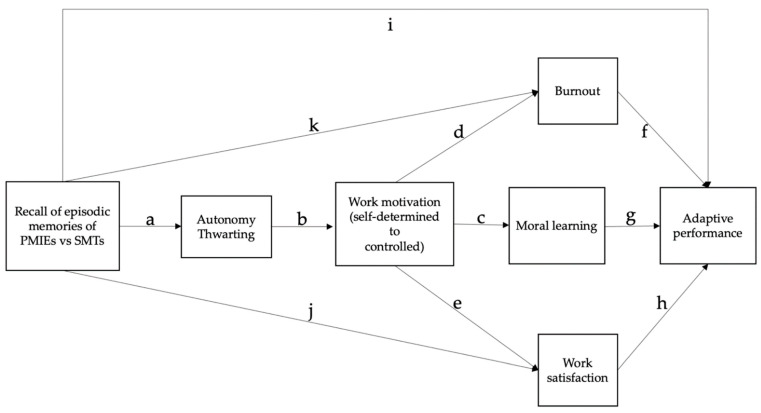
Conceptual Model of the Direct and Indirect Effects of PMIE recall on Burnout, Work Satisfaction and Adaptive Performance.

Our main aim is to examine the long-term consequences of nurses’ exposure to PMIEs during the COVID-19 pandemic on burnout, work satisfaction, and adaptive performance. Drawing from recent research on moral autobiographical memories and self-determination theory (SDT), we constructed a theoretical model (Figure 1) to investigate how memories of PMIEs affect psycho-social and occupational parameters beyond moral injury. Using an experimental design, we evaluate three paths through which memories of PMIEs may decrease nurses’ adaptive performance, with burnout, moral learning, and work satisfaction as the main mechanisms proposed (Figure 1).

PMIEs are severe moral transgressions during which individuals perceive themselves as both moral victims and moral transgressors, which is why we chose to compare them in our experiment with memories of severe moral transgressions (SMTs) during which individuals perceive themselves as moral transgressors only. Given the importance of both types of events in (re)defining nurses’ professional identities and representations of the workplace [2,23,24], our first hypothesis was that memories of SMTs and PMIEs are self-defining memories, highly important, and central to the self (H1).

Below, we present the three paths through which memories of PMIEs during the COVID-19 pandemic (as compared to memories of SMTs) might predict lower adaptive performance in nurses (Figure 1) and refer to previous results to support our hypotheses. To avoid repetitiveness, the relationships common to all three paths (i.e., between recalling PMIEs, autonomy thwarting, and work motivation), along with the direct influence of recalling PMIEs on adaptive performance are presented only for the first path.

1. Path 1: Burnout as the Main Mechanism

Our second hypothesis was that autonomy would be more thwarted in episodic memories of PMIEs than SMTs (H2; path a in Figure 1), because SMTs are intentional acts, whereas PMIEs are perceived as coerced [2,23]. As exposure to PMIEs was associated with higher burnout [25] independently of autonomy thwarting, we expected a unique differential contribution of type of memory on burnout as well (path k in Figure 1). Studies so far did not investigate how exposure to PMIEs might impact adaptive performance, to our knowledge. However, PMIEs can negatively impact general work performance, both proximally and more distally, in nurses and other healthcare workers [26]. Thus, we also expected a unique contribution of PMIE recall in explaining adaptive performance as compared to SMT recall (path i in Figure 1).

The differential autonomy thwarting between memories of PMIEs and of SMTs should lead to higher self-determined motivation for memories of SMTs than for memories of PMIEs, which should be followed by more controlled motivation (H3, path b in Figure 1) [9,10]. Past research showed that basic psychological need satisfaction operated as a mediator between self-defining work memories and burnout [11,12]. Because motivation is dependent on need satisfaction/thwarting, and motivation can predict burnout [21] (path d in Figure 1), we expected to find support for the conceptual relationships described in Figure 1 by the paths a*b*d. Given all the above and the fact that lower burnout can increase adaptive performance [26,27] (path f in Figure 1), our fifth hypothesis (H5) was that the type of memory recall, autonomy thwarting, and work motivation should differentially affect adaptive performance through burnout (H5; path a*b*d*f in Figure 1).

2. Path 2: Work Satisfaction as the Main Mechanism

Because exposure to PMIEs was associated with lower work satisfaction [22] independently of autonomy thwarting, we expected a unique differential contribution of type of memory on work satisfaction (path j in Figure 1). Since basic psychological need satisfaction operated as a mediator between self-defining work memories and work satisfaction [11,12], and motivation predicted work satisfaction [28] (path e in Figure 1), we expected to find support for the conceptual relationship described in Figure 1 by the path a*b*e. Given all the above and the fact that higher work satisfaction can increase adaptive performance [26,27] (path h in Figure 1), our sixth hypothesis (H6) was that the type of memory recall, autonomy thwarting, and work motivation should differentially affect adaptive performance through work satisfaction (H6; path a*b*e*h in Figure 1).

3. Path 3: Moral Learning as the Main Mechanism

While we did not find support for the fact that memories of PMIEs can predict the increase in moral learning found for SMTs [7], we know that thwarted autonomy could impair learning from failures by leading to more controlled work motivation [10,14] (path b*c in Figure 1. Thus, we hypothesized that the differences between memories of PMIEs and SMTs in autonomy thwarting and work motivation would lead to differences in moral learning (H4; path a*b*c in Figure 1). Given all the above and the fact that a higher number of upward counterfactuals without a moral valence increased adaptive performance [29] (path g in Figure 1), our seventh hypothesis was that the type of memory recall, autonomy thwarting, and work motivation should differentially affect adaptive performance through moral learning (H7; path a*b*c*g in Figure 1).

Overall, memories of PMIEs should impair adaptive performance more than recalling SMTs.

## 2. Materials and Methods

### 2.1. Sample

#### 2.1.1. Participants Recruiting

We conducted an experimental study on a convenience sample of nurses working in hospitals across Romania during February 2022. The data was collected after a fourth wave of the COVID-19 pandemic had a catastrophic impact on the Romanian healthcare system, leading to a sharp increase in infection and mortality, with more than 500 daily deaths and close to 20,000 daily new cases, for a population of 19 million inhabitants [30]. Given the unpreparedness of the medical system to handle this crisis and based on past results obtained in previous waves in Romania [5], we expected that nurses in all health specialties may have been exposed to morally challenging work events, often amounting to PMIEs.

For the purposes of this research, 608 nurses were contacted via e-mail and/or phone and invited to participate in our study. They were also asked to forward the invitation to other fellow nurses meeting the criteria for inclusion in our study: having worked as a nurse during the COVID-19 pandemic in a hospital for more than 6 months. Phone numbers and addresses were collected for previous research conducted by the authors when participants consented to be contacted for future research. Out of the 608 nurses contacted, 590 confirmed their availability, and 106 nurses, invited by the participants, e-mailed or messaged us to confirm their willingness to participate as well. Upon randomization in the two experimental conditions (memories of PMIEs and SMTs), we sent all 696 participants online questionnaires created in Google Forms. We received 654 complete answers and eliminated 16 participants who failed the attention checks from both experimental conditions (10 from the SMT condition and 6 from the PMIE condition). Also, we excluded 24 participants from the PMIE condition who did not recall a PMIE, according to their answers on the Moral Injury Events Scale.

#### 2.1.2. Final Sample Description

Our final sample included 614 nurses (85.3% identifying as female and 14.7% as male, with ages ranging from 21 to 57 years (*M* = 38.1, *SD* = 8.6) and with an overall work experience of *M* = 12.7 years (*SD* = 8.29). Concerning education, 91.2% of our participants had completed post-secondary studies, with 5% of them having completed bachelor’s studies and 3.7% having had a master’s degree. While all our participants worked in hospital settings, their specialties were diverse, with 13% working in Palliative Care, 12.7%—in Oncology, 10.5%—in Internal Medicine, 10.4%—in Surgery, 8.5%—in Emergency Rooms, 8.5%—in Neurology, 7%—in Psychiatry, 6.7% in Intensive Care Units, 6.4%—in Infectious Diseases, 6%—in Pneumology, 3.9%—in Obstetrics-Gynecology, 2.8%—in Hematology, 2.6%—in Gastroenterology, 1%—in Radiology and 0.2%—in Dentistry.

The final number of participants in the PMIE condition was 297. The final number of participants in the SMT condition was 317. To test our conceptual model, which estimated 37 parameters (Figure 1), a sufficient sample size would comprise 370 participants, according to the criteria of [31], who stated that the ideal goal for Structural Equation Modelling was to have a 20 to 1 ratio for the number of participants to the number of model parameters, but a ratio of 10 to 1 was acceptable, if the sample size exceeded 200. With 614 participants, our sample is closer to the ideal ratio (740) than to the acceptable ratio (370).

#### 2.1.3. Ethics

Our research adhered to the ethical guidelines outlined in the Declaration of Helsinki and was approved by the ethics committee of our faculty. All participants were over 18 and were instructed about their voluntary involvement and data confidentiality concerns. Specifically, given the sensitive nature of the data requested (episodes of severe past moral violations at their workplace), we assured participants that their anonymity would be kept and none of their data would be made public or shared with anyone other than the two main investigators (i.e., the first two authors). We adopted this policy due to our participants raising issues that they may face drastic consequences if their identities were discernable. The data collected was securely stored by the two first authors for statistical analysis. As a reward for their participation, five cash prizes of 100 RON were offered through a draw.

### 2.2. Procedure and Instruments

Data was collected with an online survey which comprised, in order, the following: informed consent, socio-demographic information, experimental task (presented in detail in Appendix A), the Moral Injury Events Scale, three items for manipulation check, two items to assess the autonomy thwarting component of their memories, two items assessing the personal importance and centrality of the memories to the self, one item to assess moral learning, the Work Extrinsic and Intrinsic Motivation Scale, the Adapted Satisfaction with Life Scale, the Emotional Exhaustion sub-scale of the Maslach Burnout Inventory, the Adaptive Performance Scale and an attention check.

The study was self-paced. After reading and agreeing with the informed consent, participants filled in socio-demographic information concerning the socio-cultural gender with which they identified, their age, and their job experience, as previous research showed that being younger, having more experience, and identifying as a woman fosters adaptive performance [29,32]. Then, following [6,8], we presented all participants with definitions and examples for the roles of “moral victims”, “moral transgressors”, and for PMIEs. Participants in the SMT condition recalled and described a work event during which they felt like moral transgressors which occurred during the COVID-19 pandemic, while participants in the PMIE condition recounted an event during which they felt like both moral victims and transgressors from the same period. For more details on the experimental procedure, please see Appendix A.

Then, we administered the 9-item Moral Injury Events Scale (MIES) modified to assess PMIEs among healthcare workers during the COVID-19 pandemic [33] (e.g., “I acted in a way that violated my own moral code or values in this instance”). The scale was tested and used on Romanian healthcare workers [5]. Answers ranged from 1—“Strongly Agree” to 6—“Strongly Disagree”. To assess whether memories were perceived as PMIEs, we dichotomized the total scores, with responses of “Moderately Agree” to “Strongly Agree” on any of the 9 items coded as exposure to a PMIE [33], excluding participants not recalling PMIEs.

All participants were asked to provide their moral judgement on the events recalled (“How morally wrong was your behavior in this instance?”), from 1—“Slightly Morally Wrong” to 7—“Very Morally Wrong” [7]. As a manipulation check, we asked participants to which extent they perceived themselves as moral victims and transgressors in those situations. Answers to the two items ranged from 1—“Not at All” to 7—“Very Much”.

The autonomy thwarting component of their memories was assessed with two items (e.g., “I felt free to do things and to think how I wanted”), with answers ranging from −3—“Strongly Disagree” to 3—“Strongly Agree”, and 0-“Do Not Agree nor Disagree/Not Applicable”. To reflect need thwarting, items were reversed, and scores were averaged [11,12]. The internal consistency of the scale was good (Cronbach’s alpha = 0.817). The Alpha Cronbach value found by Philippe et al. [11] was 0.84.

We measured with one item each the personal importance and centrality of the events to the self [8,13]: “How important is the event to you personally (it involves an important episode in your life)?” 1—”Not at All Important” to 7—“Very important”; “Is the event in your memory a central part of your life story?” 1—“Not at All Central” to 7—“Very central”. Other phenomenological characteristics of the memories not relevant were assessed, but not analyzed here.

Moral learning was measured as the frequency of morally upward counterfactual thinking [7], with the question: “Since it happened, how often have you thought about or talked about morally better ways in which you could have acted?” (1-“Never” to 7-“Very Often”).

Self-determined work motivation was assessed with the Work Extrinsic and Intrinsic Motivation Scale (WEIMS) [34]. The scale evaluates six types of motivation with three items each, reflecting the continuum of self-determination: intrinsic motivation (e.g., “Because I derive much pleasure from learning new things.”), integrated regulation (e.g., “Because it has become a fundamental part of who I am.”), identified regulation (e.g., “Because this is the type of work, I chose to do to attain a certain lifestyle.”), introjected regulation (e.g., “Because I want to succeed at this job, if not I would be very ashamed of myself.”), external regulation (e.g., “Because this type of work provides me with security.”) and amotivation (e.g., “I don’t know why, we are provided with unrealistic working conditions.”). Answers to items range from 1-“Does Not Correspond at All” to 7-“Corresponds Exactly”. The reliability of the sub-scales was acceptable, with Cronbach’s alpha coefficients greater than 0.7 (0.935 for intrinsic motivation, 0.819 for integrated motivation, 0.771 for identified motivation, 0.848 for introjected motivation, 0.808 for external motivation and 0.960 for amotivation). The Alpha Cronbach’s values found by [34] were 0.80 for intrinsic motivation, 0.83 for integrated motivation, 0.67 for identified motivation, 0.70 for introjected motivation, 0.77 for external motivation and 0.64 for amotivation. In accordance with SDT [10] and with SDT research [11], we computed the final scores with the following weighting procedure: (intrinsic × 3) + (integrated × 2) + (identified × 1) − (introjected × 1) − (external × 2) − (amotivation × 3). Higher scores reflected more self-determined work motivation, while lower scores − more controlled work motivation. Instrument reliability was also acceptable, with a Cronbach’s alpha of 0.841, very similar to the one found by Tremblay et al. [34], of 0.84.

Work satisfaction was measured with the 5-item Adapted Satisfaction with Life Scale [35,36], (e.g., “I am satisfied with the type of work I do.”), with individual answers ranging from 1-“Strongly Disagree” to 7-“Strongly Agree”. Reliability was good (Cronbach’s alpha = 0.879). The Cronbach’s Alpha coefficient value found by Bérubé et al. [35] was 0.87. Higher total scores indicated greater work satisfaction.

Burnout was assessed with the 8-item Emotional Exhaustion sub-scale of the Maslach Burnout Inventory [37], (e.g., “I feel emotionally drained from my work.”) with answers from 0-“Never” to 6-“Every Day”, adapted for Romanian healthcare providers, with a Cronbach’s alpha of 0.88 [38]. High scores indicate higher burnout. The reliability was good (Cronbach’s alpha = 0.927).

Adaptive performance was measured with the 19-item scale developed by Charbonnier-Voirin and Roussel [39] (e.g., “I develop new tools and methods to resolve new problems”), with responses from 1-“Strongly Agree” to 7-“Strongly Disagree”. Higher total scores indicated higher adaptive performance. The scale was reliable according to the Cronbach’s alpha of 0.946 we computed, greater than the ones obtained by Charbonnier-Voirin and Roussel [39] on their two different samples: 0.84 and, respectively, 0.88.

We employed the attention check used by Stanley et al. [7]: “Do you feel that you paid attention, avoided distractions, and took the survey seriously? Participants were assured that their answers would not affect their participation and prize draw or their opportunity to participate in future studies and they were asked to choose from among one of the following: 1-“No, I was distracted”; 2-“No, I had trouble paying attention”; 3-“No, I did not take this study seriously”; 4-“No, something else effected my participation negatively”; 5-“Yes”. Only participants who selected “5” were included in our analysis.

### 2.3. Data Analyses Strategy

Data analyses were conducted in Jamovi 2 (The jamovi group, Sydney, Australia) and R (R Core Team, Vienna, Austria). To test our model (Figure 1), we employed path analysis, a subset of Structural Equation Modelling used to estimate and assess direct, indirect, and mediation relationships between variables [31]. Path analyses simultaneously run sets of regression equations to determine parameter estimates and model fit. The most commonly employed estimation method is Maximum Likelihood (ML), but its estimated standard errors are less reliable when the model includes non-normally distributed, ordinal (moral learning, in our model), or categorical variables (experimental condition, in our model) [40]. The distributions of our endogenous variables departed significantly from normality (adaptive performance: *W* = 0.99, *p* = 0.004; burnout: *W* = 0.98, *p* < 0.001; work satisfaction: *W* = 0.99, *p* < 0.001; work motivation: *W* = 0.99, *p* < 0.001; autonomy: *W* = 0.95, *p* < 0.001). The diagonally weighted least-squares estimation method (DWLS, or robust WLS) generates more accurate results for ordinal, categorical, and/or non-normally distributed variables (e.g., [40]), which is why we employed it using lavaan [41]. The DWLS method does not require large samples, with 200–300 participants sufficing for accurate assessments (e.g., [40]). With 614 participants and 37 estimated parameters, employing the DWLS method further enhanced the accuracy of parameter estimation for our data. Other hypotheses were explored with Pearson correlations, Independent Samples *t*-tests, and General Linear Models.

## 3. Results

We checked our experimental manipulation and tested whether memories of PMIEs and memories of SMTs differed in terms of moral severity, perceived moral transgressor status and perceived moral victim status, as judged by the participants. Our results showed that there were no significant differences between the perceived moral severity of the recalled PMIEs (*M* = 5.58, *SD* = 1.11) and recalled SMTs (*M =* 5.51, *SD* = 1.14), *t*_(612)_ = −0.67, *p* = 0.499, Cohen’s *d* = 0.054, 95% CI [−0.10; 0.21]. Also, there were no significant differences in perceived moral transgressor status between the participants who recalled PMIEs (*M* = 5.55, *SD* = 1.11) and the ones who recalled SMTs (*M =* 5.51, *SD* = 1.14), *t*_(612)_ = −0.343, *p* = 0.732, Cohen’s *d* = −0.028, 95% CI [−0.19; 0.13]. Participants who recalled PMIEs perceived themselves as having higher moral victim status (*M* = 4.99, *SD* = 1.47) than those who recalled SMTs (*M =* 2.02, *SD* = 0.83): Welch’s *t*_(459)_ = −30.6, *p* < 0.001, Cohen’s *d* = −2.49. These results supported the equivalence between the two experimental groups in terms of perceived moral severity.

### 3.1. Socio-Demographic Differences

To assess whether nurses’ burnout, work satisfaction, thwarted autonomy, moral learning, work motivation, and adaptive performance varied with age, experience, gender, and education, we ran Pearson’s correlations, Independent Samples *t*-tests and One Way ANOVAs.

*Age and Work Experience.* Participants’ age was positively correlated with autonomy thwarting, burnout, and work experience: the older our participants were, the more they felt their autonomy thwarted during the experiences recalled and the more work experience they had (Table 1). Negative correlations with participants’ age were found for work motivation, work satisfaction, and adaptive performance: the younger the nurses, the more self-determined their motivation, the higher their work satisfaction and their adaptive performance.

Participants’ work experience was significantly associated with work satisfaction, the older the nurses’, the less satisfied with their work. We found weak correlations between autonomy thwarting, moral learning, work motivation, burnout, and adaptive performance, with more experience being associated with more autonomy thwarting and less moral learning upon PMIE and SMT recall, more burnout, and less adaptive performance.

*Gender.* Nurses identifying as female learned significantly more from these experiences from a moral standpoint as compared to nurses identifying as males (Table 2). Nurses identifying as female had significantly higher levels of adaptive performance and work motivation than nurses identifying as male (Table 2). We also found differences in work satisfaction and burnout close to reaching statistical significance, showing that nurses identifying as male experienced more burnout and less work satisfaction as compared to the ones identifying as female (Table 2).

*Education*. Education significantly influenced work motivation, with post-hoc tests showing that nurses with bachelor’s degrees were significantly more satisfied with their work as compared to nurses with post-secondary studies (Table 3).

### 3.2. Self-Defining Memories of PMIEs and SMTs

As hypothesized in H1, memories of both PMIEs and SMTs were quite important (*M_PMIE_ =* 5.19; *M_SMT_ =* 4.44) and quite central to the self (*M_PMIE_ =* 5.4; *M_SMT_ =* 4.73), since the means indicate ‘important’ and ‘rather important’ on the 1 to 7 scales used [13].

### 3.3. Path Analysis of the Conceptual Model

Although some correlations between variables (Figure 1) were strong (Table 4), VIF values were under 5, and Tolerance values were over 0.2 (Table 4 and Table A1, in Appendix B), while skewness and kurtosis were between −3 and 3 (Table 4). For information about outliers, please consult Appendix C and Table A2 and Table A3. Since multicollinearity was not problematic and internal consistency was acceptable, we ran the path analysis with DWLS estimation and percentile bootstrapping using 10,000 resamples on our data.

Our exogenous variables were “Experimental Condition” and socio-demographic characteristics for which we controlled. Our endogenous variables comprised the proposed mediators (“Autonomy Thwarting”, “Work Motivation”, “Moral Learning”, “Burnout” and “Work Satisfaction”) and our dependent variable, “Adaptive Performance” (Figure 1). Our model included both serial (“Experimental Condition → Autonomy Thwarting → Work motivation → Moral Learning”) and parallel mediation (“Moral Learning → Burnout → Adaptive Performance” and, respectively, “Moral Learning → Work Satisfaction → Adaptive Performance”). A direct path from “Experimental Condition” to “Adaptive Performance” was also included (i).

We controlled for the effects of our participants’ age on autonomy, work motivation, adaptive performance, and work satisfaction, as well as for the effects of gender on work motivation, moral learning, and adaptive performance. The variable “Experimental Condition” was dummy coded, so that the reference group was the one who recalled SMT events. This way, we could interpret path coefficients relative to the group having recalled SMTs, and make inferences regarding memories of PMIEs, the main focus of our investigation. Residual covariances between exogenous variables (“Gender”, “Age”, and “Experimental Condition”) were included in the model. Consequently, the regression equations included in our model were:

Autonomy Thwarting ← a * Experimental Condition + Age

Work Motivation ← b * Autonomy Thwarting + Experimental Condition + Age + Gender

Moral Learning ← c * Work Motivation + Autonomy Thwarting + Experimental Condition + Gender

Work Satisfaction ← e * Work Motivation + Autonomy Thwarting + j * Experimental Condition + Age

Burnout ← d * Work Motivation + Autonomy Thwarting + k * Experimental Condition

Adaptive Performance ← f * Burnout + g * Moral Learning + h * Work Satisfaction + Work Motivation + Autonomy Thwarting + i * Experimental Condition + Age + Gender

Direct and indirect effects were estimated by multiple regression analyses, with unstandardized coefficients B and standardized coefficients β for all variables. Standard Errors (SEs), Test Statistics (z-values), and *p*-values (*p*>|z|) were computed based on the unstandardized coefficients, and we assessed significance based on the 95% Confidence Intervals computed for the Standard Errors with the 10,000 re-draws percentile bootstrapping procedure. To assess the hypothesized mediation relationships, we modelled (a). Direct effects for Adaptive Performance (i), Burnout (k), and Work Satisfaction (j); indirect effects (by multiplying the path coefficients connecting the independent variables to their proposed outcomes); (c). total effects (the sum of direct and indirect effects) (Table 5). A good model fit would involve that the model converges, resulting in CFI and TLI values exceeding 0.950, SRMR values below 0.05, RMSEA values below 0.08, GFI values of over 0.95, AGFI values of above 0.9, and a CMIN/df below 3, with a non-significant chi-square test [31].

In our case, lavaan’s algorithm converged. We found a good model fit, suggesting that our specified paths may correspond to the observed data we collected (CFI = 0.999, TLI = 0.997, GFI = 1; AGFI = 1; SRMR = 0.019, RMSEA = 0.021, 95% CI [0.00; 0.05]; χ^2^(8) = 10.08, *p* = 0.259; χ2/df = 1.26). The *R*^2^ values suggested that the model accounted for 11% of the variance in autonomy thwarting (*R*^2^ = 0.109), 37.2%—in Work Motivation (*R*^2^ = 0.372), 42%—in Burnout (*R*^2^ = 0.42), 44%—in Moral Learning (*R*^2^ = 0.44), 39.7%—in Work Satisfaction (*R*^2^ = 0.397) and 66.7%—in Adaptive Performance (*R*^2^ = 0.667).

The parameter estimation confirmed our hypotheses regarding the effects of recalling PMIEs as compared to recalling SMTs on autonomy thwarting (H2), Work Motivation (H3), and Moral Learning (H4), as illustrated in Table 5. Thus, autonomy thwarting was significantly higher in memories of PMIEs than in memories of SMTs (H2). Memories of PMIEs were followed by more controlled work motivation when accounting for the contribution of autonomy thwarting as compared to memories of SMTs (H3). Recalling PMIEs was followed by lower moral learning than recalling SMTs, with a lower frequency of morally upward counterfactuals for the PMIE group than for the SMT group, when accounting for autonomy thwarting and work motivation (H4). Also, work motivation significantly predicted work satisfaction when accounting for autonomy thwarting, and for the difference between the two experimental conditions (Table 5). PMIE recall was associated with less work satisfaction as compared to SMT recall when accounting for work motivation and autonomy thwarting. Work motivation was negatively associated with burnout, and participants recalling PMIEs had higher levels of burnout and lower levels of adaptive performance than participants recalling SMTs. Adaptive performance was significantly predicted by autonomy thwarting, work motivation, work satisfaction, moral learning, and burnout, with lower burnout being associated with less autonomy thwarting, higher moral learning, and higher adaptive performance—with higher moral learning, work satisfaction, and work motivation. The estimates for age—one of our control variables—were significant only in predicting work satisfaction, with younger nurses experiencing more of it as compared to their older counterparts (Table 5). Participants identifying as female reported higher work motivation and moral learning than participants identifying as male (Table 5).

Mediation analyses were run to test H5, H6, and H7 (Table 6). Recalling PMIEs led to higher levels of burnout than recalling SMTs, a relationship mediated by autonomy thwarting and work motivation: participants recalling PMIEs had experienced more autonomy thwarting and more controlled work motivation, which led to higher burnout, in comparison to participants recalling SMTs. The indirect effect of the experimental condition on work satisfaction was also significant, with participants having recalled PMIEs, and having experienced more autonomy thwarting and more controlled work motivation, leading to less work satisfaction. Recalling PMIEs decreased nurses’ adaptive performance more as compared to recalling SMTs, following the three mediational paths proposed. First, recalling PMIEs was associated with more autonomy thwarting, leading to more controlled work motivation, higher burnout, and, consequently, lower adaptive performance. Also, recalling PMIEs decreased adaptive performance as compared to recalling SMTs through autonomy thwarting, more controlled work motivation, and less moral learning. Finally, PMIE recall resulted in lower adaptive performance than SMT recall, by thwarting autonomy, leading to more controlled work motivation and less work satisfaction (Table 6). In conclusion, H5, H6, and H7 were confirmed.

## 4. Discussion

The COVID-19 pandemic affected nurses the most in terms of physical and mental health, respectively occupational well-being, and work performance [1,2,3,4]. Moral injury is the consequence of exposure to PMIEs, which has soared during this time due to individual, social and organizational factors [5,24,42]. At the individual level, we could mention the initial lack of theoretical and procedural medical knowledge about the new coronavirus, conflicts about prioritizing personal and family health versus prioritizing patient care, as well as moral conflicts between patients’ rights to freedom versus the public health demand for their isolation [43,44]. At the social level, moral stressors included frequent personnel displacement leading to poor coordination among medical teams and divergent opinions on treatment plans, along with a perceived lack of competence of colleagues and fears that they were not respecting safety standards [43]. Organizationally, institutional unpreparedness was reflected in insufficient PPE, time, and personnel [42]. This left nurses with feelings of guilt and shame stemming from their perceived inability to save sufficient lives and protect themselves and their families [1,2,3,4]. PMIEs and moral injuries were traditionally researched in war veterans, and thus insufficiently explored in healthcare and, specifically, in nurses [23]. By drawing upon recent studies on moral autobiographical memories [6,7,8] and self-determination theory [9,10,11,12], our study explored how memories of PMIEs can impact the psycho-social functioning, mental health, and adaptive performance of nurses, thus highlighting a previously unexplored area of long-term effects of the COVID-19 pandemic.

We tested the fit of a conceptual model describing the mechanisms through which memories of PMIEs may affect nurses’ adaptive performance by increasing burnout and decreasing work satisfaction (Figure 1). Our findings show that exposure to PMIEs, autonomy thwarting, work motivation, burnout, moral learning, and work satisfaction can independently and jointly decrease nurses’ adaptive performance. Despite its relevance during the current pandemic, the study of adaptive performance in healthcare has been largely neglected [45]. Adaptive performance is one of the most important dimensions of work performance in constantly changing environments. The rapid spread of the new coronavirus created unprecedented pressure, stress, and radical practice transformations in global healthcare systems, largely unprepared to handle a health crisis of this magnitude (e.g., [3]). According to our results, exposure to work-related PMIEs could have dramatically affected this ability in nurses, emphasizing the necessity of organizations to engage in moral repair after PMIE exposure [46]. Notably, to our knowledge, this is also the first study to show that moral learning can affect nurses’ adaptive performance as well, adding to the calls to provide them with an ethically safe climate [42].

Burnout and work satisfaction were also negatively influenced by recalling PMIEs through the proposed mediational paths (Figure 1). The COVID-19 pandemic exerted a great toll on these occupational health parameters in nurses, with high levels of burnout and low work satisfaction deemed as urgent issues in healthcare [20]. Our results suggest that exposure to PMIEs, along with autonomy thwarting and controlled work motivation may have contributed to this increase, in line with previous results [11]. However, we found that work motivation mediates the relationship between the autonomy thwarting component of PMIE memories and burnout, respectively work satisfaction, which has not been shown before, to our knowledge. Therefore, our findings highlight the importance of cultivating self-determined motivation in nurses to increase job satisfaction and decrease burnout, which could also lower turnover intentions and heightens affective commitment [47], both problematic areas during the pandemic [48].

According to our findings and previous literature [10], organizational support for nurses’ autonomy could improve their self-determined motivation, and thus capitalize on their full potential [49]. Consequently, nurses should be supported in taking pride in their work, given more autonomy, encouraged to voice their opinions, and acknowledged as an invaluable part of the medical team [49].

Nurses recalling PMIEs perceived themselves as moral transgressors and as moral victims, whereas nurses recalling SMTs mainly saw themselves as moral transgressors, in line with previous studies and theoretical perspectives which emphasize that PMIEs are moral transgressions performed unwillingly, as they violate personal/professional moral values [2,23]. Since memories of PMIEs and SMTs could constitute self-defining memories according to our findings, an assumption that should be further tested, they could negatively affect how nurses perceive their work environment and their profession [11,12,13].

Furthermore, self-defining memories anchor work identities [11], and memories of PMIEs could lead to developing a sense of an immoral self-concept more than memories of SMTs, due to the perceived lack of agency during PMIEs, which impaired moral learning in our sample. When people remember a severe moral wrongdoing, they learn from it by mentally simulating alternative ways of action, which would have made them feel as if they were morally good (i.e., upward moral counterfactuals) [7]. In turn, this leads to strong intentions to behave differently in the future (i.e., moral improvement), which ensures a future morally good self-concept. The differences in moral learning that emerged in our results suggest that while this process occurred for nurses who recalled SMTs, in line with previous research [7], it did not follow the experiences of PMIEs. Then, having experienced a PMIE may alter both the present and the future morally good self-concept in nurses, essential for a positive professional identity [50]. The consequences of this alteration may extend beyond occupational self-concept and outcomes (i.e., burnout, work satisfaction, and adaptive performance) and reach the personal self-concept, since morality is an essential component of self-identity [51]. Furthermore, they could also lead to other negative health outcomes, as observed in members of armed forms exposed to PMIEs, including self-harm, suicidality, substance use, social problems, and increased risk of PTSD and depression (e.g., [23]). Future studies should examine these assumptions empirically.

Our findings are also relevant for policymakers. Supporting nurses’ autonomy organizationally and including them in the decision-making processes could have beneficial effects on the perceived impact of exposure to PMIEs [42]. Preparing nurses beforehand by providing them with an honest, direct account of the incoming ethical difficulties might decrease the risk of subsequent mental health problems [4]. Routine briefings on PMIEs, organized between peers or including a supportive supervisor, may help to reframe how they recall those incidents and their deleterious effects. Finally, healthcare organizations should engage in moral repair, re-building trust for nurses having witnessed severe moral transgressions perpetrated by their superiors [46]. After the crisis passes, staff should be actively monitored to identify members who are suffering and refer them to services of psychological assistance, where they may get the necessary help to alleviate their guilt, shame, and other psychological symptoms [4].

Our research is not without limitations. Our sample comprised an unequal number of nurses identifying as female and nurses identifying as male. Although the ratio in our sample is fairly representative of the one found in the general population of nurses in Romania [52] and around the world [53], which comprised only approximately 10% of nurses identifying as male, thus increasing our external validity, it may have influenced our results. Future studies which have a primary goal of gender differences in adaptive performance should address this when choosing their participants. Also, we had few participants with bachelor’s and master’s degrees in our samples, which is also fairly representative for nurses in Romania. However, in other contexts, this may constitute a limit to the generalizability of our results in this regard. Although considered adequate by some, our sample may be considered modest by others, so future studies could increase the number of participants when investigating this topic. Finally, due to ethical considerations, we could not provide a thematic analysis of the incidents recalled by our participants, as they would not volunteer information that could have negative consequences for them if made public, especially when recounting SMT transgressions, which could also include medical errors. However, past research has shown that self-reported judgements of morality are very reliable, with people finding it easy to detect what constitutes an (im)moral act [54]. Given that we checked our experimental manipulations, we are confident in our results in this regard. Future research should also investigate whether perceptions of intentionality play a part in autonomy thwarting for PMIEs, since past research showed that unintentional offenses are perceived as less negative [6]. It could be that PMIEs perceived as devoid of intentionality do not have such a strong impact on burnout, work satisfaction, or adaptive performance. For instance, if a fellow nurse stayed at home after contracting the new coronavirus and a patient died because of a shortage of staff, the lack of intentionality characterizing the incident lessens the psychological impact on the nurse who remembers this incident.

## 5. Conclusions

Nurses have suffered greatly from exposure to PMIEs during the COVID-19 pandemic. This was due to higher workloads and infection rates, insufficient medical supplies, and additional challenging ethical dilemmas (e.g., resource allocation when patient needs surpass available supplies), along with the guilt and shame associated with the perceived inability to save sufficient lives [1,2,4,46]. The accumulation of the moral residue left behind by PMIEs can have a detrimental effect on work performance, undermining and dehumanizing the caregiving practice. Our findings showed that unique incidents of PMIEs, when recalled, can have a negative impact on their work motivation, work satisfaction, moral learning, burnout, and adaptive performance. Other studies confirm the importance of the ethical challenges faced by nurses worldwide due to the pandemic and call for both action and more research into this phenomenon [1,2,3,5,25,42]. For instance, in the UK, self-identified burnout in NHS staff includes a significant moral component, with failure to engage in moral repair leading to long-term loss of trust and deteriorated relationships with one’s work establishment [46]. In the USA, a longitudinal study showed that nurses’ moral injury remained stable over three months in the pandemic, while psychological distress decreased, especially in unsupportive work environments [55]. In Italy and Austria, moral distress and moral injury were the main stressors with which healthcare workers were confronted, and organizational justice and decentralized decision-making were essential for mitigating their negative effects [56]. In Israel, exposure to PMIEs was high during the COVID-19 pandemic in healthcare, leading to depression, anxiety, increased self-criticism, and decreased self-compassion [57]. In China, during the COVID-19 pandemic, nurses found that organizational autonomy and connectedness support were essential for managing the wide array of ethical problems which arose [43], leading to depression, anxiety, low well-being, and emotional exhaustion [58]. In Australia, the pandemic brought about a similar host of problems, with moral stressors leading to anxiety, depression, post-traumatic stress disorder, and burnout, and with targeted interventions required to prevent or minimize exposure to PMIEs and their negative effects [44]. We join the authors above in recommending that healthcare leadership at all levels be trained to identify and prevent betrayal-based moral injury and to implement moral repair organizational practices in order to reduce turnover intentions and promote mutual trust.

## Figures and Tables

**Table 1:** Correlations between Age and Work Experience and variables of interest.

			1	2	3	4	5	6	7
**1**.	**Autonomy thwarting**	Pearson’s *r*	—						
		*p*-value	—						
		95% CI UL	—						
		95% CI LL	—						
**2**.	**Moral learning**	Pearson’s *r*	−0.53 ***	—					
		*p*-value	< 0.001	—					
		95% CI UL	−0.47	—					
		95% CI LL	−0.58	—					
**3**.	**Work motivation**	Pearson’s *r*	−0.51 ***	0.52 ***	—				
		*p*-value	< 0.001	<0.001	—				
		95% CI UL	−0.45	0.58	—				
		95% CI LL	−0.57	0.46	—				
**4**.	**Work satisfaction**	Pearson’s *r*	−0.46 ***	0.46 ***	0.52 ***	—			
		*p*-value	< 0.001	<0.001	<0.001	—			
		95% CI UL	−0.4	0.52	0.58	—			
		95% CI LL	−0.52	0.4	0.46	—			
**5**.	**Burnout**	Pearson’s *r*	0.49 ***	−0.48 ***	−0.53 ***	−0.42 ***	—		
		*p*-value	< 0.001	<0.001	<0.001	<0.001	—		
		95% CI UL	0.55	−0.42	−0.48	−0.35	—		
		95% CI LL	0.43	−0.54	−0.59	−0.48	—		
**6**.	**Adaptive performance**	Pearson’s *r*	−0.59 ***	0.64 ***	0.69 ***	0.6 ***	−0.59 ***	—	
		*p*-value	<0.001	<0.001	<0.001	<0.001	<0.001	—	
		95% CI UL	−0.54	0.68	0.73	0.65	−0.53	—	
		95% CI LL	−0.64	0.59	0.65	0.55	−0.64	—	
**7**.	**Work experience**	Pearson’s *r*	0.06	−0.02	−0.03	−0.11 **	0.01	−0.06	—
		*p*-value	0.149	0.679	0.48	0.006	0.732	0.155	—
		95% CI UL	0.14	0.06	0.05	−0.03	0.09	0.02	—
		95% CI LL	−0.02	−0.1	−0.11	−0.19	−0.07	−0.14	—
**8**.	**Age**	Pearson’s *r*	0.1 *	−0.05	−0.08 *	−0.15 ***	0.02	−0.1 *	0.83 ***
		*p*-value	0.013	0.245	0.04	<0.001	0.56	0.012	<0.001
		95% CI UL	0.18	0.03	0	−0.07	0.1	−0.02	0.85
		95% CI LL	0.02	−0.13	−0.16	−0.22	−0.06	−0.18	0.8

* p < 0.05; ** *p* < 0.01; *** *p* < 0.001; CI = confidence interval; UL = Upper Limit; LL = Lower Limit.

**Table 2:** Differences in variables of interest according to self-identified gender.

Variable	Group	Mean	*SD*	Welch’s *t*	df	*p*	Mean Difference	SE Difference	95% CI		Cohen’s *d*
									LL	UL	
**Autonomy thwarting**	M	2.73	2.6	1.36	118	0.176	0.4	0.3	−0.18	0.99	0.16
	F	2.33	2.46								
**Moral learning**	M	2.71	1.37	−3.4	126	<0.001	−0.54	0.16	−0.85	−0.22	−0.38
	F	3.25	1.45								
**Work motivation**	M	−5.73	8.53	−2.49	132	0.014	−2.48	0.99	−4.44	−0.51	−0.27
	F	−3.25	9.7								
**Work satisfaction**	M	17.78	5.28	−1.96	126	0.052	−1.19	0.61	−2.4	0.01	−0.22
	F	18.97	5.63								
**Burnout**	M	35.91	8.04	1.96	129	0.053	1.82	0.93	−0.02	3.67	0.22
	F	34.09	8.86								
**Adaptive performance**	M	67.67	17.78	−2.2	133	0.029	−4.58	2.08	−8.69	−0.47	−0.24
	F	72.25	20.57								

M = participants identifying as male (*N* = 90); F = participants identifying as female (*N* = 524); CI = confidence interval; UL = Upper Limit; LL = Lower Limit.

**Table 3:** Differences in variables of interest according to participants’ level of education.

Variable	Group ^c^	Mean	*SD*	Welch’s F	df1	df2	*p*	Games-Howell Post-Hoc Tests			*p*
								Mean Difference	*t*	df	
**Autonomy thwarting**	Post-Secondary	2.39	2.5	0.08	2	37.5	0.922				
	Bachelor’s	2.32	2.33								
	Masters’	2.57	2.25								
**Moral learning**	Post-Secondary	3.15	1.43	0.63	2	36.3	0.54				
	Bachelor’s	3.45	1.59								
	Masters’	3.3	1.61								
**Work motivation**	Post-Secondary	−3.8 ^a^	9.69	3.88	2	38.5	0.029	M ^a^ − M ^b^ = −3.79	−2.79	36	0.022
	Bachelor’s	−0.01 ^b^	7.2								
	Masters’	−3.98	8.73								
**Work satisfaction**	Post-Secondary	18.78	5.62	0.77	2	37.4	0.472				
	Bachelor’s	19.68	5.59								
	Masters’	17.87	5.01								
**Burnout**	Post-Secondary	34.38	8.85	0.09	2	37.9	0.914				
	Bachelor’s	34.29	8.67								
	Masters’	33.74	6.99								
**Adaptive performance**	Post-Secondary	71.53	20.33	0.66	2	37.2	0.524				
	Bachelor’s	74.65	18.02								
	Masters’	68.65	21.12								

^a^ The mean scores for work motivation obtained by nurses with Post-Secondary studies. ^b^ The mean scores for work motivation obtained by nurses with Bachelor’s studies. ^c^ Post-Secondary studies N = 560; Bachelor’s studies N = 31; Masters’ studies N = 23.

**Table 4:** Pearson’s Correlations and Multicollinearity Diagnostics for the Variables Included in the Tested Model.

	*M*	*SD*	Skew	Kurtosis	VIF	Tolerance	1	2	3	4	5	6
**1. Adaptive performance**	71.6	20.2	0.09	−0.36	—	—	—					
**2. Autonomy thwarting**	2.39	2.48	−0.11	−0.86	1.68	0.60	−0.59 ***	—				
**3. Moral learning**	3.17	1.45	0.25	−0.64	1.69	0.59	0.64 ***	−0.52 ***	—			
**4. Work motivation**	−3.61	9.57	−0.11	−0.63	1.94	0.52	0.69 ***	−0.51 ***	0.52 ***	—		
**5. Work satisfaction**	18.8	5.59	−0.15	−0.62	1.58	0.63	0.60 ***	−0.46 ***	0.46 ***	0.52 ***	—	
**6. Burnout**	34.4	8.77	0.21	−0.67	1.66	0.60	−0.59 ***	0.49 ***	−0.48 ***	−0.53 ***	−0.42 ***	—
**7. Experimental condition ^a^**	—	—	—	—	1.46	0.69	−0.52 ***	0.33 ***	−0.40 ***	−0.50 ***	−0.41 ***	0.44 ***

*** *p* < 0.001; ^a^ dummy coded, with 1 = PMIE and 0 = SMT.

**Table 5:** Path analysis parameter estimates.

Paths ^a^	Label	B ^d^	SE	95% CI ^b^		z	*p*	Β ^e^	H
				LL	UL				
Autonomy ← PMIE vs. SMT recall ^c^	a	1.58	0.2	1.19	1.96	8.08	<0.001	0.32	H2
Autonomy ← Age		0.02	0.01	0	0.04	1.6	0.109	0.07	
Work Motivation ← Autonomy	b	−1.44	0.14	−1.71	−1.18	−10.68	<0.001	−0.38	H3
Work Motivation ← PMIE vs. SMT recall		−6.88	0.69	−8.23	−5.51	−10.02	<0.001	−0.36	
Work Motivation ← Age		−0.01	0.04	−0.08	0.06	−0.33	0.744	−0.01	
Work Motivation ← Gender		1.87	0.92	0.04	3.71	2.03	0.043	0.07	
Moral Learning ← Work Motivation	c	0.05	0.01	0.03	0.07	5.36	<0.001	0.31	H4
Moral Learning ← Autonomy		−0.21	0.03	−0.26	−0.15	−7.42	<0.001	−0.36	
Moral Learning ← PMIE vs. SMT recall		−0.41	0.14	−0.68	−0.13	−2.98	0.003	−0.14	
Moral Learning ← Gender		0.33	0.13	0.06	0.58	2.49	0.013	0.08	
Work Satisfaction ← Work Motivation	e	0.2	0.03	0.14	0.27	6.53	<0.001	0.35	
Work Satisfaction ← Autonomy		−0.57	0.1	−0.77	−0.38	−5.79	<0.001	−0.25	
Work Satisfaction ← PMIE vs. SMT recall	j	−1.83	0.48	−2.77	−0.89	−3.8	<0.001	−0.16	
Work Satisfaction ← Age		−0.06	0.02	−0.1	−0.02	−2.9	0.004	−0.09	
Burnout ← Work Motivation	d	−0.3	0.05	−0.4	−0.21	−6.17	<0.001	−0.33	
Burnout ← Autonomy		1.03	0.17	0.69	1.36	6.08	<0.001	0.29	
Burnout ← PMIE vs. SMT recall	k	3.35	0.77	1.79	4.81	4.33	<0.001	0.19	
Adaptive Performance ← Burnout	f	−0.33	0.08	−0.49	−0.18	−4.2	<0.001	−0.14	
Adaptive Performance ← Moral Learning	g	3.19	0.52	2.2	4.23	6.13	<0.001	0.23	
Adaptive Performance ← Work Satisfaction	h	0.7	0.15	0.41	0.99	4.69	<0.001	0.19	
Adaptive Performance ← Work Motivation		0.56	0.1	0.36	0.77	5.42	<0.001	0.26	
Adaptive Performance ← Autonomy		−1.14	0.31	−1.73	−0.52	−3.68	<0.001	−0.14	
Adaptive Performance ← PMIE vs. SMT recall	i	−4.3	1.12	−6.43	−2.03	−3.83	<0.001	−0.11	
Adaptive Performance ← Age		−0.03	0.06	−0.15	0.08	−0.6	0.546	−0.02	
Adaptive Performance ← Gender		−0.41	1.32	−3.04	2.15	−0.31	0.757	−0.01	

^a^ On the left hand-side of the ←, the dependent variable in the regression, and on the right hand-side of the ←, the predictor for which coefficients were estimated. ^b^ 95% Confidence Intervals, estimated with 10,000 re-sample percentile bootstrapping for the Standard Errors. ^c^ Reference category: memories of SMTs. ^d^ Unstandardized coefficients. ^e^ Standardized coefficients. PMIE = Potentially Morally Injurious Events; SMT = Severe Moral Transgressions.

**Table 6:** Path Analysis Indirect Effects.

Effects	Label	B ^b^	SE	95% CI ^c^		z	*p*	β ^d^	H
				LL	UL				
Indirect Effect PMIE vs. SMT recall ^a^ to Adaptive Performance through Burnout	a*b*d*f	−0.23	0.07	−0.4	−0.11	−3.05	0.002	−0.01	H5
Direct Effect PMIE vs. SMT recall to Adaptive Performance through Burnout	i	−4.3	1.12	−6.43	−2.03	−3.83	<0.001	−0.11	
Total Effect PMIE vs. SMT recall to Adaptive Performance through Burnout	i + a*b*d*f	−4.53	1.12	−6.67	−2.24	−4.03	<0.001	−0.11	
Indirect Effect PMIE vs. SMT recall to Adaptive Performance through Moral Learning	a*b*c*g	−0.34	0.1	−0.55	−0.18	−3.54	<0.001	−0.01	H7
Direct Effect PMIE vs. SMT recall to Adaptive Performance through Moral Learning	i	−4.3	1.12	−6.43	−2.03	−3.83	<0.001	−0.11	
Total Effect PMIE vs. SMT recall to Adaptive Performance through Moral Learning	i + a*b*c*g	−4.64	1.13	−6.77	−2.37	−4.11	<0.001	−0.12	
Indirect Effect PMIE vs. SMT recall to Adaptive Performance through Work Satisfaction	a*b*e*h	−0.32	0.09	−0.53	−0.17	−3.46	0.001	−0.01	H6
Direct Effect PMIE vs. SMT recall to Adaptive Performance through Work Satisfaction	i	−4.3	1.12	−6.43	−2.03	−3.83	<0.001	−0.11	
Total Effect PMIE vs. SMT recall to Adaptive Performance through Work Satisfaction	i + a*b*e*h	−4.62	1.13	−6.75	−2.36	−4.1	<0.001	−0.11	
Indirect Effect PMIE vs. SMT recall to Burnout	a*b*d	0.69	0.16	0.41	1.04	4.3	<0.001	0.04	H5
Direct Effect PMIE vs. SMT recall to Burnout	k	3.35	0.77	1.79	4.81	4.34	<0.001	0.19	
Total Effect PMIE vs. SMT recall to Burnout	k + a*b*d	4.04	0.73	2.55	5.42	5.53	<0.001	0.23	
Indirect Effect PMIE vs. SMT recall to Work Satisfaction	a*b*e	−0.46	0.1	−0.68	−0.29	−4.59	<0.001	−0.04	H6
Direct Effect PMIE vs. SMT recall to Work Satisfaction	j	−1.83	0.48	−2.77	−0.89	−3.81	<0.001	−0.16	
Total Effect PMIE vs. SMT recall to Work Satisfaction	j + a*b*e	−2.3	0.46	−3.2	−1.4	−5.03	<0.001	−0.21	

^a^ Reference category: memories of SMTs. ^b^ Unstandardized coefficients. ^c^ 95% Confidence Intervals, estimated with 10,000 re-sample percentile bootstrapping for the Standard Errors. ^d^ Standardized coefficients.

## Data Availability

The data presented in this study are partially available on request from the corresponding author. The data are not publicly available due to ethical constraints created by the sensitive topic investigated, which precludes us from sharing the content of the memories of moral transgressions recalled by participants, as well as information based on which participants could be identified.

## Appendix A. Experimental Procedure

The study was self-paced. After reading and agreeing with the informed consent, participants filled in socio-demographic information concerning the socio-cultural gender with which they identified, their age and their job experience, as previous research showed that being younger, having more experience and identifying as a woman fosters adaptive performance [29,59,60]. Then, following [6,8], we provided definitions and examples for the roles of “moral victims” and “moral transgressors” to participants in both experimental conditions. Moral transgressors were defined as “individuals whose intentions and actions bring about harmful events” and moral victims as “individuals who experience feelings and emotions brought about by the moral transgressor’s actions” [6]. We also informed participants that the same individual can be a moral transgressor or a moral victim at different times or even at the same time. We adapted the examples of moral transgressors and victims used by the authors in their study to better fit nurses’ work environment and to reflect more severe moral transgressions. We operationalized the severity of moral transgressions by the magnitude of the harmful effects it had on the patient [61]. The transgressions used as examples were devised according to Brüggemann et al. [62].

“Laura is a nurse at a hospital in Romania. One morning during the 4th wave of the COVID-19 pandemic, she woke up feeling sick, and tested herself for COVID-19 at home with three rapid tests. Although all three tests were positive, she went to work anyway, because she had the opportunity to work an extra shift and make more money. Patients and colleagues contracted the infection from her and several of them are still in the ICU, with reserved prognoses. Laura felt terribly guilty and ashamed about the consequences of her action”.

Then, they were presented with a different example, which flipped the moral victim/transgressor roles of the first example:

“Laura is a nurse at a hospital in Romania. During the 4th wave of the COVID-19 pandemic, she unknowingly cared for a patient who was infected with COVID-19. The patient knew about the infection, but lied about it, taking advantage of the fact that patients were not tested prior to being committed. Laura, along with several other patients and colleagues, contracted COVID-19 from the patient, and she is now in the ICU, with a reserved prognosis. Laura felt betrayed and angered about the consequences of the patient’s action”.

Finally, we presented them with a definition of PMIEs: “events or action during which you felt as both a moral victim and a moral transgressor, when you did or witnessed something you felt was morally wrong not because you wanted to, but because you felt as if you did not have a choice” [2,23]. We then followed with an example of PMIE reflecting an issue with which many nurses and healthcare providers in Romania were confronted during the 4th wave of the pandemic:

“Laura is a nurse at a hospital in Romania. During the 4th wave of the COVID-19 pandemic, the beds in the ICU were all occupied, and she had to care for a patient in the ER. The patient was rapidly deteriorating, and he badly needed access to a ventilator. None were available, and the necessary procedure for transferring the patient to another hospital were stalled due to bureaucracy issues. The patient’s oxygen saturation dropped quickly, and, despite Laura’s and the doctor’s best efforts, he died before the papers for the transfer were ready. Laura felt both guilty for not having saved the patient’s life and betrayed by the medical system which allowed for this to happen”.

All the examples presented to the participants referenced work-related situations in which the main actants were nurses and patients, both for the purposes of equivalency between the two experimental conditions (*episodic memory of SMT* and *episodic memory of PMIE*) and to prepare our participants to recall memories central to their main work activity—caring for patients.

“Please describe a personal memory of a specific event related to your work during the COVID-19 pandemic in which you were a moral transgressor, as defined and exemplified above. Select a memory significant to you which is at least three-month-old, and which often comes to your mind. This memory should be of the most morally wrong thing you have done during the pandemic with harmful consequences on a patient. Describe in a general fashion what happened, where it happened, who you were with (if anyone), and how you and other people reacted. Please remember we are not interested in the identities of anybody involved, so feel free to use phrases such as ‘a colleague’, ‘a boss’, ‘a patient’, and other generic denominators. What is important to us is for you to remember specific details, not for us to know them. Describe your role and what have been the consequences of your reaction or of your actions during this event. Please provide enough details so that we can fully understand what happened, as if you were telling a story to someone. We would also like to assure you that the content of your memories will not be shared with anybody outside of the two first authors and it will not be used in our analyses”.

Participants in the PMIE condition received the same instruction, with the first sentence modified as such: “Please describe a personal memory of a specific event related to your work during the COVID-19 pandemic in which you were both a moral transgressor and a moral victim, a PMIE, as defined and exemplified above”.

## Appendix B

**Table A1:** Collinearity diagnostics.

Predictors	Outcome Variables											
	Adaptive Performance		Burnout		Work Satisfaction		Moral Learning		Work Motivation		Autonomy Thwarting	
	VIF	T	VIF	T	VIF	T	VIF	T	VIF	T	VIF	T
**Autonomy Thwarting**	1.68	0.59	1.37	0.73	1.37	0.73	1.37	0.73	1.13	0.88		
**Moral Learning**	1.71	0.59										
**Work Motivation**	1.94	0.52	1.62	0.62	1.62	0.62	1.62	0.62	1.13	0.89		
**Work Satisfaction**	1.6	0.63										
**Burnout**	1.67	0.6										
**Experimental Condition ^a^**	1.46	0.68	1.34	0.75	1.34	0.75	1.34	0.75			1.01	0.99
**Age**	1.03	0.97			1.01	0.99			1.01	0.99	1.01	0.99
**Gender**	1.02	0.98					1.01	0.99	1	0.99		

*Note*: T = Tolerance values. ^a^ dummy coded, with 1 = PMIE and 0 = SMT.

## Appendix C. Outliers Treatment

To detect multivariate outliers, we employed Cook’s Distance Test [63], to evaluate the influence of outliers on the regression coefficients by measuring the change in parameter estimates when extreme cases are deleted. Usually, Cook’s values greater than one are considered influential and eliminated from the dataset [64]. A more restrictive criterion is eliminating the observations with a Cook’s distance over four times the mean [62,63]. With *M =* 0.00190 (*SD =* 0.01), the computed reference value for our model was 0.0076. We did not find any observation with a Cook’s value over 1. However, 28 observations exceeded the reference value, ranging from 0.139 to 0.007.

We excluded them from the analysis and ran the model again. We did not find differences in parameter and effect estimations compared to the model including the 28 cases (Table A2 and Table A3), so we decided to keep the initial results (Table 5 and Table 6), in order to retain the advantage of having a larger dataset. The model fit was slightly improved after removing the outliers (CFI = 1, TLI = 0.998, GFI = 1; AGFI = 1; SRMR = 0.019, RMSEA = 0.018, 95% CI [0.02; 0.05]; χ2(8) = 9.56, *p* = 0.297; χ2/df = 1.195). The *R*^2^ values suggested that the model accounted for 12.2% of the variance in autonomy thwarting (*R*^2^ = 0.122), 39%—in Work Motivation (*R*^2^ = 0.390), 43.7%—in Burnout (*R*^2^ = 0.437), 45.9%—in Moral Learning (*R*^2^ = 0.459), 44.9%—in Work Satisfaction (*R*^2^ = 0.449) and 71.2%—in Adaptive Performance (*R*^2^ = 0.712).

**Table A2:** Path analysis parameter estimates after outlier removal.

Paths ^a^	Label	B ^d^	SE	95% CI ^b^		z	*p*	Β ^e^	H
				LL	UL				
Autonomy ← PMIE vs. SMT recall ^c^	a	1.65	0.2	1.27	2.03	8.44	<0.001	0.34	H2
Autonomy ←Age		0.02	0.01	−0.00	0.05	1.77	0.077	0.08	
Work Motivation ← Autonomy	b	−1.37	0.13	−1.63	−1.11	−10.39	<0.001	−0.36	H3
Work Motivation ← PMIE vs. SMT recall		−7.32	0.67	−8.62	−5.98	−10.86	<0.001	−0.39	
Work Motivation ← Age		−0.02	0.04	−0.09	0.05	−0.57	0.571	−0.02	
Work Motivation ← Gender		2.09	0.94	0.28	3.93	2.22	0.026	0.08	
Moral Learning ← Work Motivation	c	0.05	0.01	0.03	0.07	5.51	<0.001	0.33	H4
Moral Learning ← Autonomy		−0.21	0.03	−0.26	−0.15	−7.3	<0.001	−0.36	
Moral Learning ← PMIE vs. SMT recall		−0.36	0.14	−0.62	−0.08	−2.53	0.012	−0.12	
Moral Learning ← Gender		0.26	0.13	0	0.51	1.94	0.052	0.06	
Work Satisfaction ← Work Motivation	e	0.25	0.03	0.19	0.31	8.25	<0.001	0.42	
Work Satisfaction ← Autonomy		−0.57	0.09	−0.75	−0.38	−6.05	<0.001	−0.25	
Work Satisfaction ← PMIE vs. SMT recall	j	−1.34	0.45	−2.2	−0.44	−2.97	0.003	−0.12	
Work Satisfaction ← Age		−0.05	0.02	−0.09	−0.01	−2.38	0.017	−0.07	
Burnout ← Work Motivation	d	−0.34	0.05	−0.44	−0.24	−6.5	<0.001	−0.36	
Burnout ← Autonomy		1.06	0.18	0.71	1.4	6.06	<0.001	0.3	
Burnout ← PMIE vs. SMT recall	k	2.66	0.81	1.03	4.2	3.3	0.001	0.15	
Adaptive Performance ← Burnout	f	−0.32	0.07	−0.46	−0.19	−4.61	<0.001	−0.14	
Adaptive Performance ← Moral Learning	g	3	0.41	2.21	3.81	7.42	<0.001	0.22	
Adaptive Performance ← Work Satisfaction	h	0.63	0.11	0.41	0.85	5.5	<0.001	0.18	
Adaptive Performance ← Work Motivation		0.62	0.08	0.47	0.79	7.71	<0.001	0.3	
Adaptive Performance ← Autonomy		−1.02	0.24	−1.47	−0.55	−4.32	<0.001	−0.13	
Adaptive Performance ← PMIE vs. SMT recall	i	−4.27	1.01	−6.2	−2.23	−4.22	<0.001	−0.11	
Adaptive Performance ← Age		−0.03	0.05	−0.13	0.08	−0.53	0.593	−0.01	
Adaptive Performance ← Gender		−1.12	1.13	−3.4	1.07	−1	0.320	−0.02	

^a^ On the left hand-side of the <-, the dependent variable in the regression, and on the right hand-side of the <-, the predictor for which coefficients were estimated. ^b^ 95% Confidence Intervals, estimated with 10,000 re-sample percentile bootstrapping for the Standard Errors. ^c^ Reference category: memories of SMTs. ^d^ Unstandardized coefficients. ^e^ Standardized coefficients.

**Table A3:** Path Analysis Indirect Effects after outlier removal.

Effects	Label	B ^b^	SE	95% CI ^c^		z	*p*	Β ^d^	H
				LL	UL				
Indirect Effect PMIE vs. SMT recall ^a^ to Adaptive Performance through Burnout	a*b*d*f	−0.24	0.07	−0.41	−0.12	−3.31	0.001	−0.01	H5
Direct Effect PMIE vs. SMT recall to Adaptive Performance through Burnout	i	−4.27	1.01	−6.2	−2.23	−4.22	<0.001	−0.11	
Total Effect PMIE vs. SMT recall to Adaptive Performance through Burnout	i + a*b*d*f	−4.52	1.01	−6.44	−2.47	−4.51	<0.001	−0.12	
Indirect Effect PMIE vs. SMT recall to Adaptive Performance through Moral Learning	a*b*c*g	−0.35	0.09	−0.55	−0.2	−3.81	<0.001	−0.01	H7
Direct Effect PMIE vs. SMT recall to Adaptive Performance through Moral Learning	i	−4.27	1.01	−6.2	−2.23	−4.22	<0.001	−0.11	
Total Effect PMIE vs. SMT recall to Adaptive Performance through Moral Learning	i + a*b*c*g	−4.62	1.02	−6.57	−2.57	−4.55	<0.001	−0.12	
Indirect Effect PMIE vs. SMT recall to Adaptive Performance through Work Satisfaction	a*b*e*h	−0.35	0.1	−0.56	−0.19	−3.67	<0.001	−0.01	H6
Direct Effect PMIE vs. SMT recall to Adaptive Performance through Work Satisfaction	i	−4.27	1.01	−6.2	−2.23	−4.22	<0.001	−0.11	
Total Effect PMIE vs. SMT recall to Adaptive Performance through Work Satisfaction	i + a*b*e*h	−4.62	1.02	−6.57	−2.56	−4.54	<0.001	−0.12	
Indirect Effect PMIE vs. SMT recall to Burnout	a*b*d	0.76	0.17	0.46	1.14	4.43	<0.001	0.04	H5
Direct Effect PMIE vs. SMT recall to Burnout	k	2.66	0.81	1.03	4.2	3.3	0.001	0.15	
Total Effect PMIE vs. SMT recall to Burnout	k + a*b*d	3.62	0.76	1.89	4.88	4.52	<0.001	0.2	
Indirect Effect PMIE vs. SMT recall to Work Satisfaction	a*b*e	−0.56	0.11	−0.80	−0.37	−5.1	<0.001	−0.05	H6
Direct Effect PMIE vs. SMT recall to Work Satisfaction	j	−1.34	0.45	−2.2	−0.44	−2.98	0.003	−0.12	
Total Effect PMIE vs. SMT recall to Work Satisfaction	j + a*b*e	−1.9	0.43	−2.74	−1.05	−4.43	<0.001	−0.17	

^a^ Reference category: memories of SMTs. ^b^ Unstandardized coefficients. ^c^ 95% Confidence Intervals, estimated with 10,000 re-sample percentile bootstrapping for the Standard Errors. ^d^ Standardized coefficients.
